# Long‐Term Implant Survival in Periodontitis

**DOI:** 10.1111/clr.70105

**Published:** 2026-02-15

**Authors:** Almir Kashta, Sebastian‐Edgar Baumeister, Benjamin Ehmke, Stefan Lars Reckelkamm

**Affiliations:** ^1^ Clinic for Periodontology and Conservative Dentistry University of Münster Münster Germany; ^2^ Institute of Health Services Research in Dentistry University of Münster Münster Germany

**Keywords:** dental implants, osseointegration, peri‐implantitis, periodontitis, survival analysis

## Abstract

**Objectives:**

This study assessed whether periodontitis stage and grade are associated with long‐term implant survival in periodontally compromised patients.

**Methods:**

A retrospective analysis was conducted among 362 periodontitis patients who received 1106 implants at the University Clinic Münster, Germany, with a median follow‐up of 85.5 months (range: 3.6–236.4). Periodontitis stage and grade were assessed based on clinical and radiographic data at the first clinical visit. Kaplan–Meier curves and Cox regression models were used to analyze implant survival.

**Results:**

A total of 85 implants (7.69%) failed, with nearly half of these losses occurring within the first six months (failed osseointegration). Cumulative survival rates were 95.8% at 60 months, 91.1% at 120 months, and 82.6% at 180 months. Grading, but not staging, was significantly associated with implant loss. Patients classified as grade C had a higher risk of implant loss at any given time during follow‐up than patients with grade A or B (hazard ratio = 2.78; 95% confidence interval: 1.18–6.54).

**Conclusions:**

Our findings demonstrate that periodontitis, particularly in terms of grading as defined by the current classification, is a significant risk factor for implant loss, even in patients undergoing periodontal therapy. Both clinicians and patients should consider these risk factors during treatment planning, as they can substantially impact implant survival. Nevertheless, dental implants remain a reliable and predictable therapeutic option, even for patients with severe periodontitis.

## 1 Introduction

Tooth loss is an important oral health issue. Depending on the location and extent, it causes functional impairment with regard to chewing and aesthetics and ultimately affects quality of life by limiting dietary options and reducing social engagement (Gerritsen et al. 2010; Fleming et al. 2020). Global estimates place the prevalence of edentulism at 4.1%, with 353 million people affected (Nascimento et al. 2024). One of the main reasons for tooth loss is periodontitis, a chronic inflammatory disease associated with dysbiotic plaque biofilms and characterized by progressive destruction of the tooth‐supporting apparatus (Pihlstrom et al. 2005; Papapanou et al. 2018).

The use of dental implants for the rehabilitation of partially or fully edentulous patients constitutes a safe, accepted, and commonly applied treatment (Tomasi and Derks 2022). Nevertheless, patients receiving implants after tooth loss due to periodontitis face a higher risk of complications, such as lack of osseointegration, peri‐implant diseases, and ultimately implant loss (Renvert and Persson 2009; de Ry et al. 2021).

Several potential risk indicators for these adverse outcomes, such as smoking, poor oral hygiene, and diabetes mellitus also represent risk factors for periodontitis (Tomasi and Derks 2022; Moy et al. 2005; Genco and Borgnakke 2013). They are further used to define periodontal diagnosis, describing disease “Staging” for its extent, severity, and complexity as well as “Grading” for its progression rate (Papapanou et al. 2018).

To date, only a few studies have considered the role of periodontal staging and grading on implant success (Heitz‐Mayfield et al. 2020; Yamazaki et al. 2022; Ravidà et al. 2021). Hence, this study aims to assess the relationship between periodontal diagnosis and long‐term survival of implants in a cohort of periodontitis patients.

## 2 Materials and Methods

**FIGURE 1:**
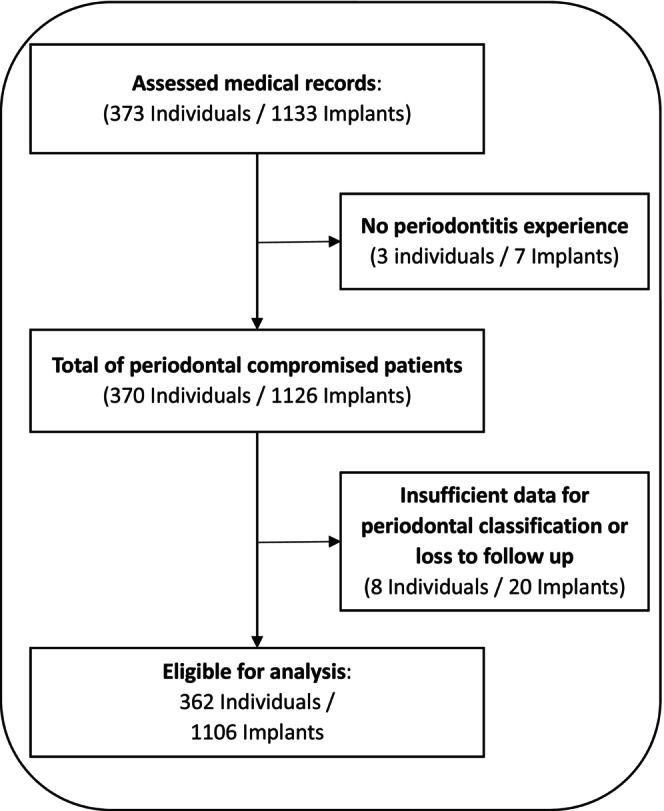
Study participants flow chart.

For data analysis, dental records of patients treated at the Department of Periodontology and Restorative Dentistry at the University Clinic Münster, Germany, were reviewed. The evaluation included patients who had received at least one dental implant placed by a single periodontist (BE) since the introduction of digital medical records in 2005. Exclusion criteria were no periodontitis experience or insufficient data for periodontal classification (see Figure 1). Periodontal staging and grading was determined according to the current classification of periodontal diseases and conditions based on the clinical and radiographic examinations at first visit (Tonetti and Sanz 2019). Risk factors, such as diabetes mellitus and smoking, were also recorded and considered for the periodontal diagnosis. Personally identifying variables were removed before analysis.

Prior to implantation, all patients underwent comprehensive periodontal therapy including anti‐infective treatment and, when indicated, surgical periodontal procedures. This was followed by supportive periodontal care, during which the implants were placed. However, the final treatment course depended on patient compliance and adherence, as well as external circumstances. Two distinct implant systems, Xive and Straumann, were used. If necessary, bone augmentation was performed using autogenous bone. Implants were loaded after a healing period of 3–6 months, following a conventional loading protocol. Initial examination date, time of implant insertion, patients' age, implant position, and time of implant loss were extracted from dental records. If no loss occurred, the time of the last examination in our clinic was recorded.

This manuscript was prepared in accordance with STROBE (Strengthening the Reporting of Observational Studies in Epidemiology) guidelines for reporting observational studies (von Elm et al. 2008).

### 2.1 Statistical Analysis

Implant survival was defined as a binary variable at implant level. Each implant has been classified as either present or explanted. Accordingly, implant survival time refers to the time between implant placement and either explantation or loss to follow‐up. Early implant loss was further defined as a survival time of less than six months following placement (Tomasi and Derks 2022). The time‐to‐event data was analyzed using Kaplan–Meier survival estimates. We visualized overall and group‐stratified survival using Kaplan–Meier curves. Group differences were assessed with Cox proportional hazards models that included a patient‐level random effects (shared frailty) term to account for clustering of implants within patients. We fitted univariable frailty Cox models for each factor of interest and a multivariable frailty Cox model including periodontitis grade & stage, age, and sex as covariates. Results are reported as hazard ratios (HR) with 95% confidence intervals (CI) (Austin 2017). The proportional hazards assumption was assessed using Schoenfeld residuals. To evaluate robustness to potential informative censoring, we conducted a sensitivity analysis in which we constructed extreme‐case scenarios by assuming that all censored implants either survived or failed at the censoring time and additionally modelled intermediate scenarios in which 10%–50% of censored implants were assumed to fail within 12 months after dropout. We also examined differences between periodontitis staging and grading groups within a healthy subgroup restricted to nonsmoking, nondiabetic participants. The analyses were performed using the survival (3.5‐5), survminer (0.4.9), frailtypack (3.5.1) and ggplot2 (3.4.4) packages in R, version 4.3.0.

### 2.2 Ethics

The information utilized originates from the Münster Clinic for Periodontology's medical (dental) records. The relevant local ethical review bodies granted corresponding ethics approval (2023‐485‐f‐S) and raised no fundamental ethical or legal concerns regarding the conduct of the study. Informed consent was not obtained in accordance with Paragraph 32 of the Declaration of Helsinki, as the study involved a retrospective analysis of anonymized patient data.

## 3 Results

### 3.1 Study Cohort and Patient Characteristics

Between January 1, 2005, and December 31, 2022, a total of 362 patients meeting the inclusion criteria were identified from the database as having received at least one dental implant. Among these patients were 182 women (50.3%) and 180 men (49.7%), with a mean age of 63.46 ± 9.62 years, ranging from 33 to 90 years. Within the cohort, 10.5% had known diabetes, and 11.6% reported being current or former smokers. In total, 1106 implants were placed. On average, women received slightly fewer implants (3.03 implants per individual) than men (3.08 implants per individual). Additional baseline characteristics at both the patient and implant level are provided in Table 1.

**TABLE 1:** Study sample characteristics.

	Implant level	Patient level
Number of implants/patients	1106	362
Female	551 (49.8)	182 (50.3)
Age	64.29 (9.76)	63.46 (9.62)
Stage (%)		
1	48 (4.3)	21 (5.8)
2	181 (16.4)	73 (20.2)
3	601 (54.3)	205 (56.6)
4	276 (25.0)	63 (17.4)
Grade (%)		
A	168 (15.2)	69 (19.1)
B	458 (41.4)	161 (44.5)
C	480 (43.4)	132 (36.5)
Ever‐smoker	135 (12.2)	42 (11.6)
Diabetes	127 (11.5)	38 (10.5)

*Note:* Data are presented as mean (SD) or absolutes (percentages).

### 3.2 Implant Survival by Periodontal Status

**FIGURE 2:**
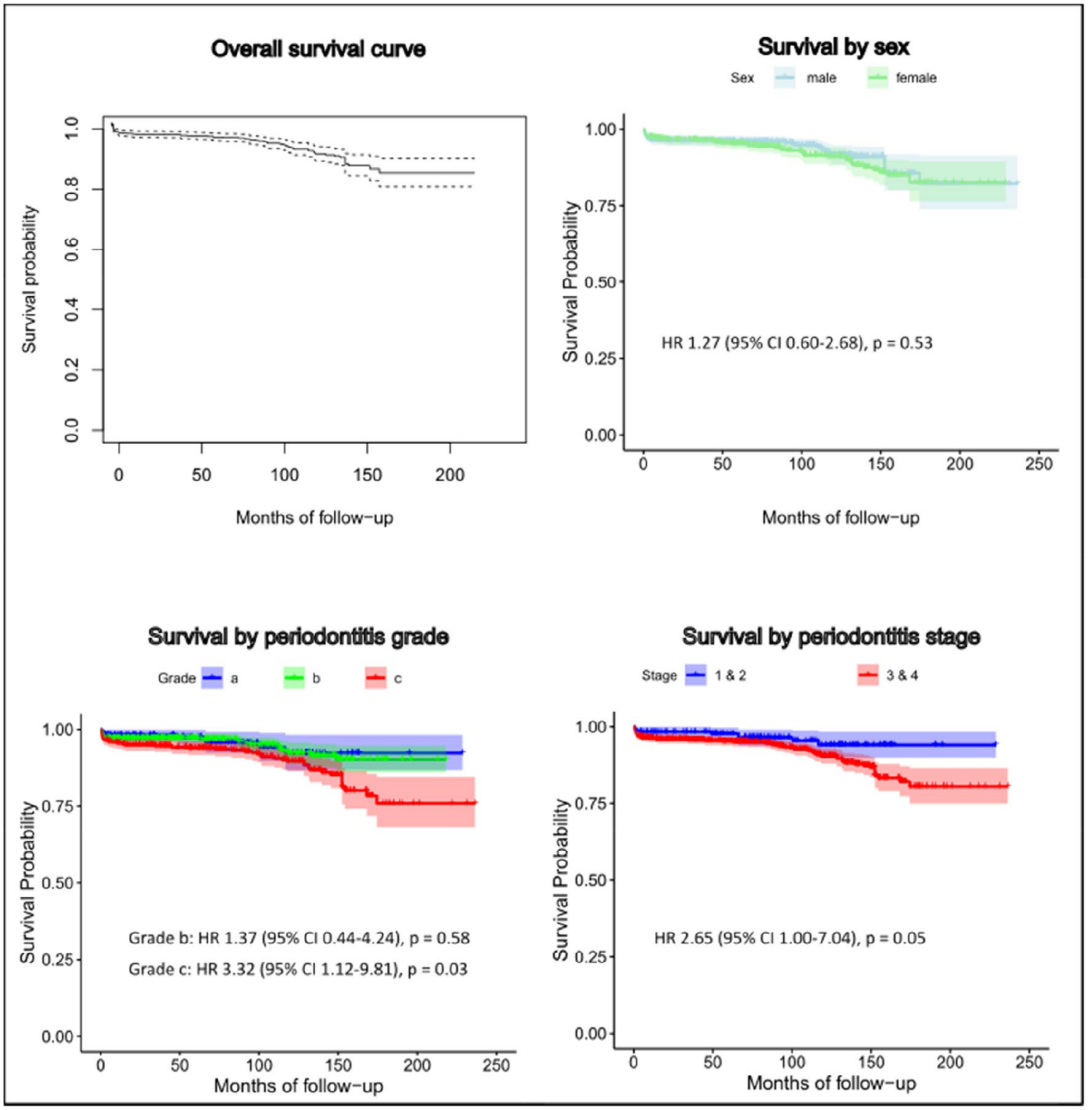
Kaplan–Meier plots for implant survival. The panels show cumulative implant survival over time. Hazard ratios (HRs) and *p*‐values (*p*) are derived from univariable Cox models with patient‐level frailty. Compared with Grade a, Grade c showed a significantly higher hazard of implant loss (HR 3.32, 95% CI 1.12–9.81; *p* = 0.03). Compared with Stage 1–2, Stage 3–4 showed a borderline‐significant increase in hazard (HR 2.65, 95% CI 1.00–7.04; *p* = 0.05).

The median observation time per patient, from the first implant insertion to the last documented visit, was 92.0 months (ranging from 3.5 to 236.4 months). On an implant‐level basis, the median observation time from implant placement to either implant loss or loss to follow‐up was 85.5 months (ranging from 0.4 to 236.4 months). Out of a total of 1106 implants, 85 were ultimately lost (7.69%). From these 85 implants, 38 (3.44%) were early implant losses (EIL). Of the 47 later losses, most were attributed to peri‐implantitis (either surgical removal or spontaneous loss). Only four implants were removed due to fracture, and one implant labeled as peri‐implantitis was found to have fractured upon removal. The cumulative survival rates were 96.6% at 12 months, 95.5% at 60 months, 91.1% at 120 months, and 82.6% at 180 months (Table 2 and Figure 2).

**TABLE 2:** Implant survival by descriptives.

	Total	Failure rate (%)	Early implant loss
Number of implants	1106	85 (7.69)	38 (3.44%)
Mean observation time in months (SD)	88.95 (54.65)	/	/
Sex			
Male	555 (50.18%)	38 (6.85%)	20 (3.6%)
Female	551 (49.82%)	47 (8.53%)	18 (3.27%)
Stage (%)			
1	48 (4.34%)	1 (2.08%)	1 (2.08%)
2	181 (16.37%)	8 (4.42%)	3 (1.66%)
3	601 (54.34%)	58 (9.65%)	27 (4.49%)
4	276 (24.95%)	18 (6.52%)	7 (2.54%)
Grade (%)			
A	168 (15.19%)	8 (4.76%)	3 (1.79%)
B	458 (41.41%)	25 (5.46%)	13 (2.84%)
C	480 (43.4%)	52 (10.83%)	22 (4.58%)
Smoking			
Ever‐smoker	135 (12.21%)	14 (10.37%)	6 (4.44%)
Never‐smoker	971 (87.79%)	71 (7.31%)	32 (3.3%)
Diabetes			
Yes	127 (11.48%)	15 (11.81%)	8 (6.3%)
No	979 (88.52%)	70 (7.15%)	30 (3.06%)

Using Cox proportional hazards models with a patient‐level shared frailty term to account for clustering, we first examined unadjusted associations between groups and implant loss. No significant association was observed between sex and implant loss (HR for females vs. males: 1.27; 95% CI: 0.60–2.68; *p* = 0.53). For periodontitis grade, Grade B did not differ significantly from Grade A (HR: 1.37; 95% CI: 0.44–4.24; *p* = 0.58), whereas Grade C showed a significantly higher hazard of implant loss at any given time during follow‐up (HR: 3.32; 95% CI: 1.12–9.81; *p* = 0.03). Patients with periodontal Stage 3–4 demonstrated a borderline‐significant increase in hazard compared with Stage 1–2 (HR: 2.65; 95% CI: 1.00–7.04; *p* = 0.05). Estimates from the multivariable Cox model indicated that patients with periodontitis grade C had a 2.78‐fold higher risk of implant loss at any given time during follow‐up compared to those with grades A and B (95% CI: 1.18; 6.64) (Table 3). In contrast, periodontal stage (95% CI: 0.55; 5.11), age (95% CI: 0.99; 1.07) and sex (95% CI: 0.61; 2.89) were not significant predictors of implant failure. Additionally, the frailty term was highly significant (variance = 4.97, *p* < 0.001), indicating substantial inter‐individual variability in the risk of implant loss. This suggests that unmeasured patient‐specific factors, such as systemic conditions or oral hygiene behaviors, contributed to implant survival beyond the included covariates.

**TABLE 3:** Frailty Cox regression model.

Characteristic	HR	95% CI	*p*
Grade			
A − B	—	—	
C	2.78	1.18–6.54	0.02
Age	1.03	0.99–1.07	0.10
Sex			
Male	—	—	
Female	1.33	0.61–2.89	0.47
Stage			
1–2	—	—	
3–4	1.67	0.55–5.11	0.37
Frailty (patient)		Var = 4.97	< 0.001

Abbreviations: CI, confidence interval; HR, hazard ratio; Var, variance.

Sensitivity analyses assuming 10%–30% of censored implants failed 12 months after dropout yielded 60‐month survival of 93%–87% versus 95.5% observed, supporting robustness to informative censoring (Figure S2 and Table S2). Further, in nonsmokers and nondiabetics, Kaplan–Meier and shared‐frailty Cox analyses showed a similar trend toward higher failure with worse periodontal status, but without statistical significance (Figure S3 and Table S3).

Overall, our results demonstrate high implant survival rates in periodontally compromised patients, with grade C exhibiting the highest risk of implant failure, both due to inadequate osseointegration and subsequent implant loss.

## 4 Discussion

In this study, we followed 362 periodontally compromised patients rehabilitated with 1106 implants over a maximum of 236 months, with a mean follow‐up of 89 months. A total of 85 implants (7.69%) failed, with nearly half of these failures occurring within the first six months, indicating early implant loss. The cumulative survival rate was 95.8% (95% CI: 94.5–97.0) at 60 months, declining to 82.6% (95% CI: 77.7–87.9) at 180 months. When considering survival in relation to periodontal status, a greater periodontal burden was associated with an increased risk of implant failure. Further analysis indicated that grading played a more decisive role in predicting implant success than staging. Nevertheless, implants remain a viable and predictable treatment option even for periodontally highly compromised patients.

Our findings align with previous research, which reported higher implant loss rates in periodontally compromised patients. Longitudinal studies have indicated cumulative survival rates of 94%–98.8% at 60 months in patients with a history of periodontitis (García‐Bellosta et al. 2010; Baelum and Ellegaard 2004). However, in cases of severe periodontitis and extended follow‐up durations, implant survival rates tend to decline further, with some studies suggesting a drop to approximately 78% after 120 months (Baelum and Ellegaard 2004).

Our data regarding EIL mirrors existing literature. Studies report that EIL occurs in approximately 2%–6% of all placed implants, accounting for 40%–83.4% of total implant failures (Tomasi and Derks 2022; Budmiger et al. 2024). However, these rates vary significantly depending on patient selection criteria and clinical settings.

Consistent with our results, prospective research indicates that implant failure is more accurately predicted by periodontal grading than staging. For example, Ravidà et al. (2021) reported that implant failure and peri‐implantitis severity were strongly associated with grading but not staging. While their study observed an increasing trend in implant failure rates from Stage I/II (0%) to Stage IV (6.5%), this trend did not reach statistical significance, suggesting that staging alone may not adequately predict implant failure. Similarly, Yamazaki et al. (2022) found no direct correlation between staging and implant loss. However, they observed a significantly higher prevalence of peri‐implantitis in Stage IV patients. Furthermore, patients who underwent bone augmentation, a procedure frequently necessary for Stage III/IV periodontitis cases, exhibited higher implant failure rates.

The success of dental implantation is largely dependent on osseointegration, which is defined as a “direct functional and structural connection between living bone and the surface of a load‐carrying implant” (Albrektsson et al. 2012). Implant failure can occur early when osseointegration fails to establish, or late when osseointegration is lost due to factors such as peri‐implantitis and progressive bone loss (Kwok et al. 2023).

Periodontal grading and staging are closely interconnected but describe distinct aspects of the disease and its management (Papapanou et al. 2018). Grading reflects host response and systemic influences, incorporating key risk factors such as diabetes and smoking, with the latter being a well‐established risk factor for peri‐implant diseases and implant failures (Tomasi and Derks 2022). Staging describes the extent of periodontal tissue destruction and structural damage already sustained due to periodontitis. Unlike grading, staging “characteristics” remain unchanged even after successful periodontal therapy. While gingival health on a reduced periodontium may pose challenges for implant placement, it does not necessarily hinder osseointegration. This may explain why staging alone does not consistently predict implant survival. In line with this rationale, analyses in a healthy subgroup restricted to nonsmokers and nondiabetics showed the same trend toward higher implant loss with worse periodontal status, but without statistical significance—likely reflecting the markedly smaller sample‐size and attenuation of exposure contrast after excluding major risk factors (see Figure S3 and Table S3).

One of the key strengths of this study is the minimization of operator‐related variability, as all implant placements were performed by a single experienced periodontologist. This approach reduces the influence of surgical skill variation and treatment planning differences, thereby enhancing the reliability of our findings. Additionally, the large sample size and long follow‐up period strengthen the study's ability to assess long‐term implant survival in periodontally compromised patients.

Nevertheless, several limitations must be acknowledged. First, as a retrospective study, we lack important data on key factors such as the reasons for loss to follow‐up or other potentially significant variables that could influence implant survival. This limitation restricts our ability to fully assess patient outcomes and identify potential confounders that may have contributed to implant failure. However, even assuming substantial informative censoring bias—specifically, that 10%–30% of censored implants failed 12 months after dropout—survival estimates remained high (60‐month survival 93%–87% vs. 96% in the original analysis; Tables S1 and S2). Second, our study population represents a convenience sample from a university‐based, real‐world setting. Although a standardized regimen was offered, the final course of therapy depended on patient preferences and adherence, as well as external circumstances—particularly COVID‐19–related interruptions of recall visits—resulting in variability in treatment regimens. Accordingly, the approach was less rigid than clinical trials with fixed treatment protocols, yet more structured than practices without a periodontal focus. This may limit the generalizability of our findings to other clinical environments, such as practices with less extensive maintenance protocols or treatment options. Another limitation is the absence of periodontally healthy patients, which prevents direct comparisons between implant survival in individuals with and without a history of periodontitis. While our study provides valuable insights into implant survival in periodontally compromised patients, future research should incorporate prospective designs with matched control groups to further elucidate the impact of periodontitis on long‐term implant success.

## 5 Conclusions

Taken together, our findings demonstrate that periodontitis, particularly in terms of grading as defined by the current classification, remains a significant risk factor for implant loss, even in a cohort following a periodontal therapy protocol. Both dental practitioners and patients should carefully consider these risk factors during treatment planning, as they may have a strong impact on implant survival. Nevertheless, dental implants remain a reliable and predictable therapeutic option, even in patients with severe periodontitis.

## Author Contributions

**Almir Kashta:** contributed to conceptualization, investigation, methodology, project administration, resources, drafting, and writing – review and editing. **Stefan Lars Reckelkamm:** contributed to conception, design, data acquisition, analysis, and interpretation, drafted and critically revised the manuscript. **Benjamin Ehmke:** provided clinical expertise and treated all patients; contributed to data curation and review of the final manuscript. **Sebastian‐Edgar Baumeister:** provided statistical guidance and contributed to the interpretation of data; reviewed and approved the final version of the manuscript. All authors gave their final approval and agreed to be accountable for all aspects of the work.

## Funding

The authors have nothing to report.

## Conflicts of Interest

The authors declare no conflicts of interest.

## Supporting information


**Data S1:** STROBE Statement—checklist of items that should be included in reports of observational studies.

**Figure S1:** Informative censoring—extreme scenarios.
**Figure S2:** Survival under varying probabilities of informative censoring.
**Figure S3:** Kaplan–Meier plots for implant survival in a healthy subgroup.
**Table S1:** Informative censoring—extreme scenarios.
**Table S2:** Survival under varying probabilities of informative censoring.
**Table S3:** Frailty Cox regression model in the healthy subgroup.

## Data Availability

Data sharing is not applicable due to the sensitivity of the underlying patient data.
