# Supplementary material for: Long‐Term Implant Survival in Periodontitis
Source: Clin Oral Implants Res. 2026 Feb 15;37(5):623–9. doi: 10.1111/clr.70105 (PMC13155307; doi:10.1111/clr.70105)
Supplement: Supplementary file 2 — Figure S1: Informative censoring—extreme scenarios. Figure S2: Survival under varying probabilities of informative censoring. Figure S3: Kaplan–Meier plots for implant survival in a healthy subgroup. Table S1: Informative censoring—extreme scenarios. Table S2: Survival under varying probabilities of informative censoring. Table S3: Frailty Cox regression model in the healthy subgroup. [file CLR-37-623-s001.docx]

**Sensitivity of survival estimates to informative censoring**

**Supplementary Figure 1: Informative censoring - extreme scenarios**


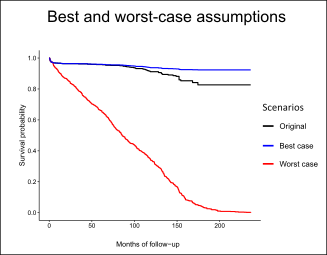


**Supp. Fig 1:** The plot shows cumulative implant survival over time under best-, worst-, and original-case assumptions. The best-case scenario assumes all censored implants survived until the end of follow-up, while the worst-case assumes all censored implants failed immediately. The original curve shows the observed Kaplan–Meier estimate without additional assumptions

**Supplementary Figure 2: Survival under varying probabilities of informative censoring**


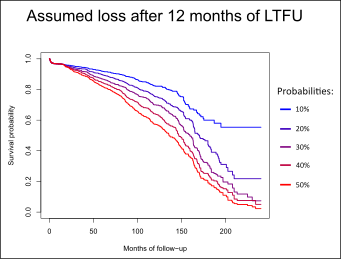


**Supp. Fig 2:** Values represent Kaplan–Meier estimates assuming that between 10% and 50% of censored implants failed 12 months post–loss to follow‑up. As the assumed failure rate rises, survival estimates decline.

**Sensitivity analysis restricted to nonsmoking, nondiabetic individuals**

**Supplementary Figure 3: Kaplan-Meier plots for implant survival in a healthy subgroup**


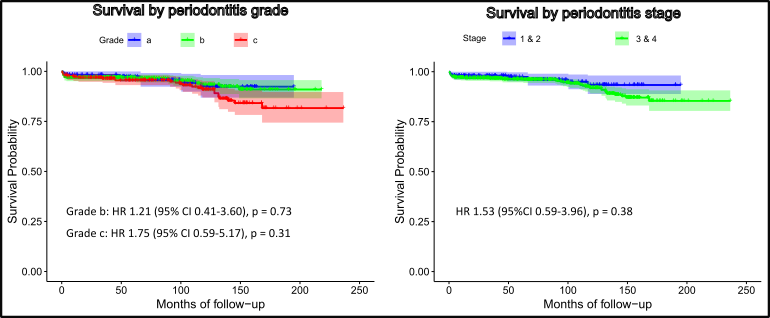


**Supp. Fig 3:** The plots depict cumulative implant survival over time in a healthy subgroup limited to nonsmoking, non-diabetic participants. The trend of limited survival by worse periodontal status is evident but not statistically significant.

**Supplementary Table 1: Informative censoring - extreme scenarios**

| **Scenario** | **12mo** | **60mo** | **120mo** | **180mo** |
| --- | --- | --- | --- | --- |
| Original | 0.97 | 0.96 | 0.91 | 0.83 |
| Worst-case | 0.91 | 0.66 | 0.34 | 0.04 |
| Best-case | 0.97 | 0.96 | 0.94 | 0.92 |

*Note:* Cumulative implant survival probabilities at 12, 60, 120, and 180 months. Original = observed estimate under the standard assumption of non-informative censoring. Worst-case = all censored cases counted as failures at the time of censoring. Best-case = all censored cases remain event-free throughout follow-up *Abbreviations:* mo = months of follow-up

**Supplementary Table 2: Survival under varying probabilities of informative censoring**

| **Failure rate** | **12mo** | **60mo** | **120mo** | **180mo** |
| --- | --- | --- | --- | --- |
| 10% | 0.97 | 0.93 | 0.82 | 0.62 |
| 20% | 0.97 | 0.90 | 0.75 | 0.41 |
| 30% | 0.97 | 0.87 | 0.70 | 0.32 |
| 40% | 0.97 | 0.84 | 0.63 | 0.25 |
| 50% | 0.97 | 0.82 | 0.59 | 0.19 |

*Note:* Cumulative implant survival probabilities at 12, 60, 120, and 180 months when a specified proportion of censored patients is assumed to experience the event exactly 12 months after censoring. *Abbreviations:* mo = months of follow-up.

**Supplementary Table 3: Frailty Cox regression model in the healthy subgroup**

| **Characteristic** | **HR** | **95% CI** | **p-value** |
| --- | --- | --- | --- |
| **Grade** |  |  |  |
| A – B | — | — |  |
| C | 1.76 | 0.66 - 4.68 | 0.3 |
| **Age** | 1.03 | 0.99 - 1.07 | 0.2 |
| **Sex** |  |  |  |
| Male | — | — |  |
| Female | 1.29 | 0.57 - 2.94 | 0.5 |
| **Stage** |  |  |  |
| 1 - 2 | — | — |  |
| 3 - 4 | 1.26 | 0.42 – 3.76 | 0.7 |
| **frailty(Patient)** |  |  | 0.006 |
| ^1^HR = Hazard Ratio, CI = Confidence Interval, Var = Variance | | | |
